# Low and mismatched socioeconomic status between newlyweds increased their risk of depressive symptoms: A multi-center study

**DOI:** 10.3389/fpsyt.2022.1038061

**Published:** 2023-01-10

**Authors:** Hong Gan, Mengdie Li, Xiaorui Wang, Qianhui Yang, Ying Tang, Baolin Wang, Kaiyong Liu, Peng Zhu, Shanshan Shao, Fangbiao Tao

**Affiliations:** ^1^Department of Maternal, Child and Adolescent Health, School of Public Health, Anhui Medical University, Hefei, Anhui, China; ^2^Key Laboratory of Population Health Across Life Cycle (Anhui Medical University), Ministry of Education of the People’s Republic of China, Hefei, Anhui, China; ^3^NHC Key Laboratory of Study on Abnormal Gametes and Reproductive Tract, Hefei, Anhui, China; ^4^Anhui Provincial Key Laboratory of Population Health and Aristogenics, Anhui Medical University, Hefei, Anhui, China

**Keywords:** newlyweds, depressive symptoms, socioeconomic status matching, education matching, income matching

## Abstract

**Background:**

While there is evidence that low socioeconomic status (SES) increases susceptibility to depression, few studies have focused on the effect of marital SES matching on depression. This study aimed to explore the impact of SES indicator matching on depressive symptoms in newlyweds and clarify the interaction of depressive symptoms between couples.

**Methods:**

We assessed the depressive symptoms of newlyweds (*N* = 28, 179 couples) using a 9-item Patient Health Questionnaire. Logistic regression models and restricted cubic splines were used to explore the association between SES indicator matching and depressive symptoms and the interaction of depressive symptoms in newlyweds, respectively.

**Results:**

Compared with newlyweds with both high-level SES, the newlyweds with both low-level SES, male higher SES, or female higher SES had an increased risk of depressive symptoms in husbands (*OR* = 1.31; 1.22; 1.30), wives (*OR* = 1.30; 1.36; 1.32), and couples (*OR* = 1.48; 1.56; 1.57) (all *P* < 0.05). In addition, as the level of depression in one partner increases, the risk of depression in the other partner also increases.

**Conclusion:**

Mismatched SES and low-level SES between couples have adverse effects on depressive symptoms in newlyweds, with depressive symptoms having a positive association between newlyweds.

## Introduction

Depression is one of the most common mental disorders, accounting for 10% of the non-fatal disease burden worldwide (1). Approximately 280 million people suffered from depression in 2019 (2). According to the China National Mental Health Development Report (2019–2020), young people aged 18–34 had the highest rates of depression among adults (3); this may be because adults at this age, especially newlyweds, face many challenges related to work, starting new families, relationships, and lifestyles. Depression among newlyweds may not only cause physical and mental health issues (4, 5) but also affect the nerve and brain development of their offspring (6, 7) since depression is associated with long-term risks of persistence (8, 9) and recurrence (10).

The interdependence theory posits that interacting partner can affect their experiences of each other, especially in terms of relatively plastic traits (e.g., psychiatric disorders) (11). Recently, a large cohort that investigated the depressive symptom trajectories of middle-aged and elderly couples found that couples in the increasing and decreasing depression classes (accounting for approximately 15% of the couples) showed synchronization in the direction of their depressive symptoms during the 12 follow-up years (12). Importantly, couples with both depressed partners are more likely to divorce and suffer bereavement (12). In addition, due to assortative mating, people usually tend to form intimate relationships with individuals similar to themselves and can be observed for similar psychological characteristics (e.g., depression) (13, 14). In other words, depression symptoms in newlyweds may affect both partners and cause comorbidities.

Low socioeconomic status (SES), despite evidence to the contrary (15), is well documented as a risk factor for depression (16–20). With increasing domestic and international inequalities in income, education, and employment (21), socioeconomic disparities in mental health are persistent (15, 16, 19, 21–23). Based on the conservation of resources (COR) theory, SES is related to the availability of resources (24). Individuals with higher education and income have more resources and are more able to protect their own resources (24). In contrast, low SES makes access to resources difficult and even hinders the conservation of resources, causing mental health problems (24). Several studies have found an association between lower material standards of living and increased depression symptoms, while improved living standards could reduce depressive symptoms (22).

Socioeconomic status indicator matching between couples can affect marriage satisfaction and happiness. In the SES model, income, employment, and educational level are not isolated entities but are linked to and affect each other. The improvement in the education level and economic status of women has enhanced their bargaining power in the family. The SES indicator matching has been found to affect the consistency of domination of family economic and social capital, thus affecting the space and resistance of communication and the emergence of conflict between couples, which is closely related to the marital quality and happiness index of couples (25). Several studies have verified the rationality of SES indicators in assortative mating: it can improve marriage satisfaction, maximize family output, and enhance marriage stability (26). Researchers have found that higher SES indicators in men can promote life satisfaction and happiness levels for either gender (27, 28). Recently, a couple-centered study found positive effects of spouse SES similarity on marital quality (29). However, few studies have focused on the impact of multiple and comprehensive SES indicator matching on depressive symptoms in individuals and couples and their interactions with newlyweds.

In the present study, we aimed to describe individual depressive symptoms and rates of depressive symptoms in couples among newlyweds and test two hypotheses based on a large newlywed cohort: (1) Low SES and the mismatch of SES between couples were associated with a risk of depressive symptoms; and (2) depressive symptoms in one partner can affect the other, especially in couples with low or mismatched SES.

## Materials and methods

### Study design

This study used data from the Reproductive Health of Childbearing Couples—Anhui Cohort (RHCC-AC), a large prepregnancy cohort study based on reproductive couples. The study aims to identify the independent and combined effects of prepregnancy lifestyles and environmental exposure on infertility, adverse pregnancy outcomes, and offspring growth and development.

**FIGURE 1:**
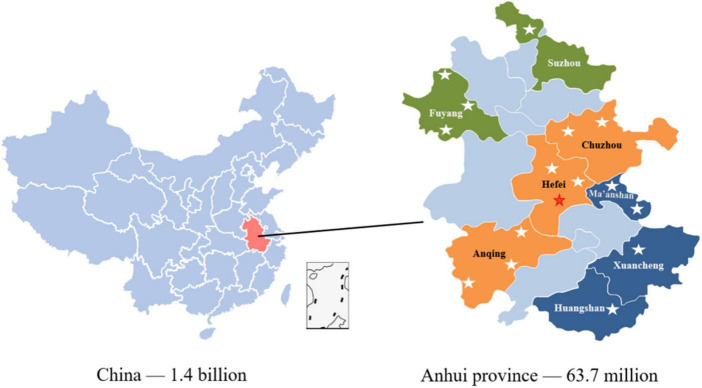
Distribution of multi-center recruitment cities/countries.

The newlywed cohort data were collected from the maternal and child healthcare family planning service center in 16 cities/counties (including one pre-survey city) in Anhui Province (140,100 km^2^, 61.13 million permanent resident population, and 4,295.92 billion Yuan in GDP) from April 2019 to June 2021. The study subcenter was selected according to the geographical distribution characteristics, economic development level, and cooperation degree of Anhui Province. The geographical locations of the 16 cities/counties in Anhui Province that participated in our cohort recruitment are shown in Figure 1. According to the geographical distribution characteristics, these 16 cities are located in the north, center, and south of Anhui Province. According to the public data of the statistical bureau of each city in Anhui Province, these 16 cities have a high and low GDP value distribution. According to the cooperation degree, these maternal and child healthcare family planning service centers have a good relationship with our research group. Inclusion criteria were (1) couples who provide informed consent, (2) couples who have never had children together, and (3) couples who can understand and complete the questionnaires independently. Exclusion criteria were (1) couples with serious organic or mental diseases and (2) couples who show poor compliance, refuse to complete the questionnaires, or are unwilling to participate in follow-up investigations. Newlyweds who met the inclusion and exclusion criteria were recruited as participants voluntarily and signed an informed consent form.

### Study population

**FIGURE 2:**
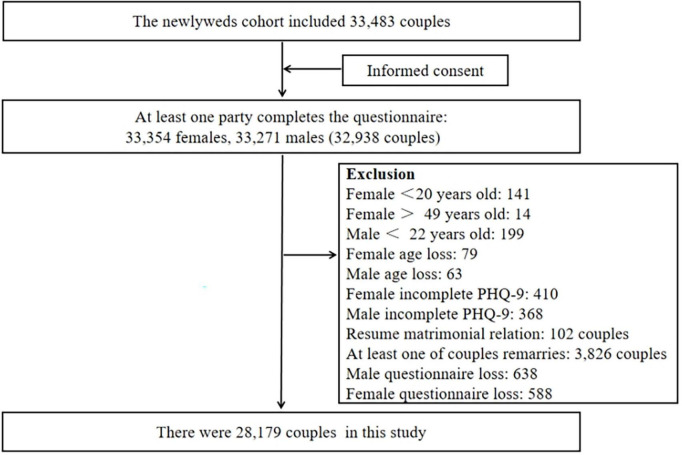
Selection of the study population. PHQ-9, 9-item Patient Health Questionnaire.

The participants were recruited from the newlywed cohort, which included 66,625 subjects (33,354 women and 33,271 men, with 32,938 couples). In the present study, people who have remarried, men aged <22 years, women aged <20 years and >49 years, and those who did not mention their age were excluded. In addition, participants who did not complete the 9-item Patient Health Questionnaire (PHQ-9) were excluded. Finally, a total of 28,179 couples were included, as shown in Figure 2.

### Socioeconomic status indicators and their matching between couples

Socioeconomic indicators were identified through self-administered questionnaires. Education was defined as the highest attained educational level and divided into the following three categories: junior high school or below (1 point), senior high school or technical secondary school (2 points), and college degree or above (3 points). Income, defined as individual annual income, was categorized as “less than 60,000 *Yuan* (1 point),” “60–100,000 *Yuan* (2 points),” and “at least 100,000 *Yuan* (3 points).” Employment for the last month was divided into the following two categories: employed (1 point) and unemployed (0 points). Education, income, and employment for the last month were combined into one variable, SES. More specifically, individuals with different levels of education, income, and employment for the last month were assigned points according to the above criteria, and the scores were summed as the SES score. Indicators for SES matching in newlyweds are shown in Table 1.

**TABLE 1:** Indicators for SES matching in newlyweds.

Variable	Explanation
**Education matching**	
Both high-level education	Both of them had a college degree or above
Both low level education	Both of them had education level less than a college degree
Male higher education	Husband’s academic qualifications was higher than wife’s
Female higher education	Wife’s academic qualifications was higher than husband’s
**Income matching**	
Both high-level income	Husband’s and wife’s incomes were at least 60,000 yuan per year
Both low-level income	Husband’s and wife’s incomes were less than 60,000 yuan per year
Male higher income	Husband’s income ranges was more than wife’s
Female higher income	Wife’s income ranges was more than husband’s
**Employment matching**	
Both unemployed	Both of them were unemployed for the last month
Both employed	Both of them were employed for the last month
Only male employed	Only the husband was employed last month
Only female employed	Only the wife was employed last month
**SES matching**	
Both high-level SES	SES scores of the husband and wife were equal and at least 6 points
Both low-level SES	SES scores of the husband and wife were equal and less than 6 points
Male higher SES	SES score of the husband was at least 1 point higher than the wife
Female higher SES	SES score of the wife was at least 1 point higher than the husband

SES, socioeconomic status.

### Depressive symptoms

We assessed the depression symptoms of participants using the PHQ-9 depression scale, which is one of the most commonly used self-administered tools for screening depression. The PHQ-9 is well-validated, with previous research showing both sensitivity and specificity of 88% for major depression compared to the results of an interview with a mental health professional (30). The PHQ-9 consists of nine items evaluating the mental and emotional states of subjects in the past 2 weeks. Each item is scored on a 4-point scale as follows: 0 (not at all), 1 (several days), 2 (more than half the days), and 3 (nearly every day). The total score ranges from 0 to 27 points (30), with a higher score indicating severe depressive symptoms. Based on the PHQ-9 score, depression symptoms can be divided into the following three categories: no depressive symptoms (0–4 points), mild depressive symptoms (5–9 points), and moderate to severe depressive symptoms (10–27 points) (30).

The individual depressive symptoms, which focus on symptoms of one partner, were divided into the following four categories: depressive symptoms of husbands (a husband with depressive symptoms regardless of the depressive symptoms in his wife), depressive symptoms of wives (a wife with depressive symptoms regardless of depressive symptoms in her husband), depressive symptoms of only husbands (a husband with depressive symptoms and his wife without depressive symptoms), and depressive symptoms of only wives (a wife with depressive symptoms and her husband without depressive symptoms). Depressive symptoms of couples determined the presence of depressive symptoms in both spouses.

### Statistical analysis

All statistical analyses were performed using SPSS 23.0 (SPSS Inc., Chicago, IL, USA) and R 3.6.1 software, and images were produced using GraphPad Prism 8.0 (GraphPad Inc., San Diego, CA, USA). The frequency and percentage (%) of the classified variables were calculated. The significance level was set at α = 0.05.

The difference in SES matching distribution between depressive symptoms in newlyweds was calculated using the chi-squared test. Univariable logistic regression models were used to detect the association between SES indicator matching and depressive symptoms in newlyweds. Multivariable logistic regression models adjusted for covariates such as age, body mass index (BMI), region, current pregnancy, and physical activity were used to explore the association. Restricted cubic splines (RCS) with 6 knots were used to flexibly model the dose-response relationship between PHQ-9 scores of one partner and depressive symptoms of the spouse (PHQ-9 score of 0–4 [no] vs. 5–27 [mild or moderate to severe]). Statistical models were adjusted for age, BMI, region, current pregnancy, and physical activity. To confirm the stability of the results, we stratified depression by severity to explore the correlation between SES indicator matching and the degree of depression. Moreover, we explored the association between marital depressive symptoms stratified by SES matching.

## Results

### Sociodemographic characteristics of the study population and distribution differences in rates of depressive symptoms

This study comprised a total of 28,179 newlyweds, with 6,457 (22.9%) wives who were currently pregnant. The average ages of men and women were 26.55 years (SD: 2.938) and 25.44 years (SD: 2.902), respectively.

**FIGURE 3:**
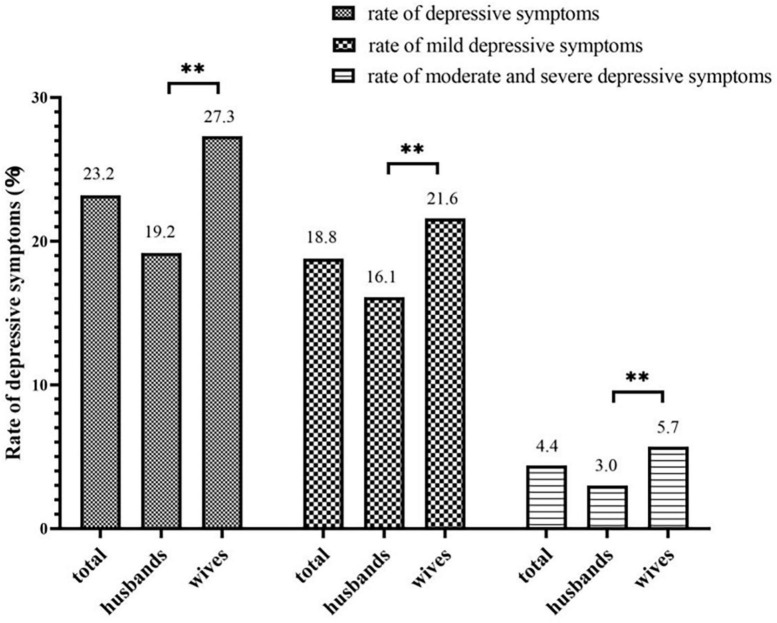
Gender differences in the detection rate of depressive symptoms. **P < 0.01.

The rate of depressive symptoms among the newlyweds was 23.2% (13,054/56,358): 18.8% suffered from mild depressive symptoms and 4.4% from moderate to severe depressive symptoms. The rate of depressive symptoms in women was significantly higher than in men (27.3 vs. 19.2%) (Figure 3). In newlyweds, the rate of depressive symptoms in both partners was 6.6%; the rate of couples with no depressive symptoms in both partners was 60.3%; the rate of depressive symptoms in only husbands was 12.4%; and the rate of depressive symptoms in only wives was 20.7%.

In husbands and wives, there were significant differences in the rate of depressive symptoms based on age, education, personal annual income, employment for the last month, region, BMI, and physical activity (*P* < 0.05). In addition, the rates of depressive symptoms were higher in wives who were currently pregnant (*P* < 0.01) (Supplementary Table 1). Furthermore, there were significant differences in the distribution of sociodemographic characteristics in SES indicator matching (Supplementary Table 2). There was a statistical difference between the matching of education, income, employment, SES, and the rate of depressive symptoms (*P* < 0.05). In newlyweds with high-level education, income homogamy, or where both partners were employed, the lowest rate of depressive symptoms was recorded in husbands and wives, both individually and as couples. Similar findings were observed for newlyweds with both high-level SES (Table 2).

**TABLE 2:** Differences in socioeconomic status matching distribution between depressive symptoms.

	*N*	Detection of depression symptoms		Couples’ depression symptoms		
		**Males (%)**	**Females (%)**	**C1 (%)**	**C2 (%)**	**C3 (%)**
**Couples**						
**Education matching**						
Both high-level education	14577	17.5	23.8	12.1	18.4	5.4
Both low-level education	4873	21.5	31.4	13.3	23.1	8.2
Male higher education	3652	20.6	32.2	12.2	23.8	8.4
Female higher education	5077	20.1	29.9	12.7	22.5	7.4
*P*-value		<0.001	<0.001		<0.001	
**Income matching**						
Both high-level income	4777	16.6	22.4	11.6	17.4	5.0
Both low-level income	7054	21.8	29.8	13.5	21.5	8.3
Male higher income	14754	18.3	28	11.9	21.5	6.4
Female higher income	1594	20.8	24.7	14.9	18.8	5.9
*P*-value		<0.001	<0.001		<0.001	
**Employment matching**						
Both unemployed	1341	20.9	31.3	12.3	22.7	8.6
Both employed	19583	18.3	25.1	12.4	19.3	5.9
Only male employed	5613	20.3	33.6	11.8	25.2	8.5
Only female employed	1108	23.7	27.3	16.1	19.7	7.7
*P*-value		<0.001	<0.001		<0.001	
**SES matching**						
Both high-level SES	3358	15.7	20.8	11.3	16.3	4.4
Both low-level SES	4912	20.6	27.5	13.9	20.8	6.7
Male higher SES	15371	18.9	28.5	12.0	21.6	6.9
Female higher SES	4004	20.4	27.3	13.5	20.3	7.0
*P*-value		0.047	<0.001		<0.001	

C1, detection rate of only husband with depressive symptoms; C2, detection rate of only wife with depressive symptoms; C3, detection rate of couples with depressive symptoms.

### Univariate logistic regression between socioeconomic status matching and depressive symptoms in newlyweds

**FIGURE 4:**
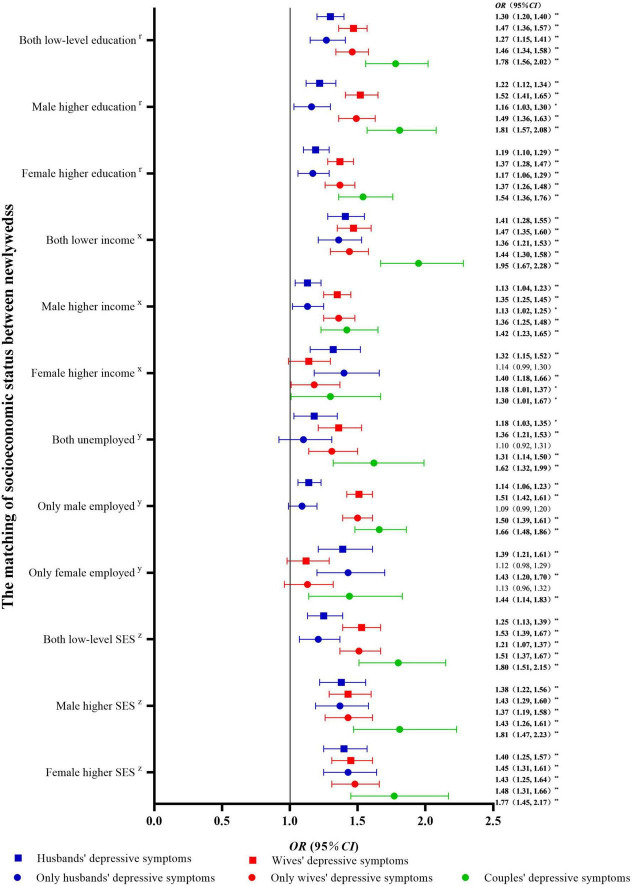
Univariable logistic regression between socioeconomic status matching and depressive symptoms in newlyweds compared with non-depressive symptoms. OR, odds ratio; CI, confidence interval; SES, socioeconomic status. ^**^P < 0.01: *P < 0.05. *^r^*Compared with newlyweds with both high-level education. *^x^*Compared with newlyweds with both high-level incomes. *^y^*Compared with newlyweds with both employed. *^z^*Compared with newlyweds with both high-level SES.

A univariate logistic regression model was used, with SES matching as the independent variable and depressive symptoms (PHQ-9 score of 0–4 [no] vs. 5–27 [mild or moderate to severe]) as the dependent variables. The results are shown in Figure 3. Compared with newlyweds with high-level education for both partners, newlyweds with low-level education for both partners, male higher education, and female higher education had an increased risk of depressive symptoms in individuals and couples (*P* < 0.05). Compared with newlyweds with a high-level income for both partners, newlyweds with a low-level income for both partners, male higher income, and female higher income had an increased risk of depressive symptoms in individuals and couples (*P* < 0.05). Furthermore, compared with newlyweds with both partners employed, newlyweds with both partners were unemployed showed an increased risk of depressive symptoms in individuals and couples. The newlyweds with only husbands employed showed an increased risk of depressive symptoms in individuals and couples. Newlyweds with only wives employed had an increased risk of depressive symptoms in husbands and couples. Compared with newlyweds with both high-level SES partners, newlyweds with both low-level SES partners, male higher SES, and female higher SES showed an increased risk of depressive symptoms in individuals and couples (*P* < 0.05) (see Figure 4 for odds ratio [*OR*] and 95% confidence interval [*CI*] values). Similar results were obtained when three classifications of depressive symptoms (PHQ-9 score of 0–4 [no] vs. 5–9 [mild] vs. 10–27 [moderate to severe]) were used as an alternative indicator of dependent variables (Supplementary Figure 1).

### Multivariable logistic regression between socioeconomic status matching and depressive symptoms in newlyweds

**FIGURE 5:**
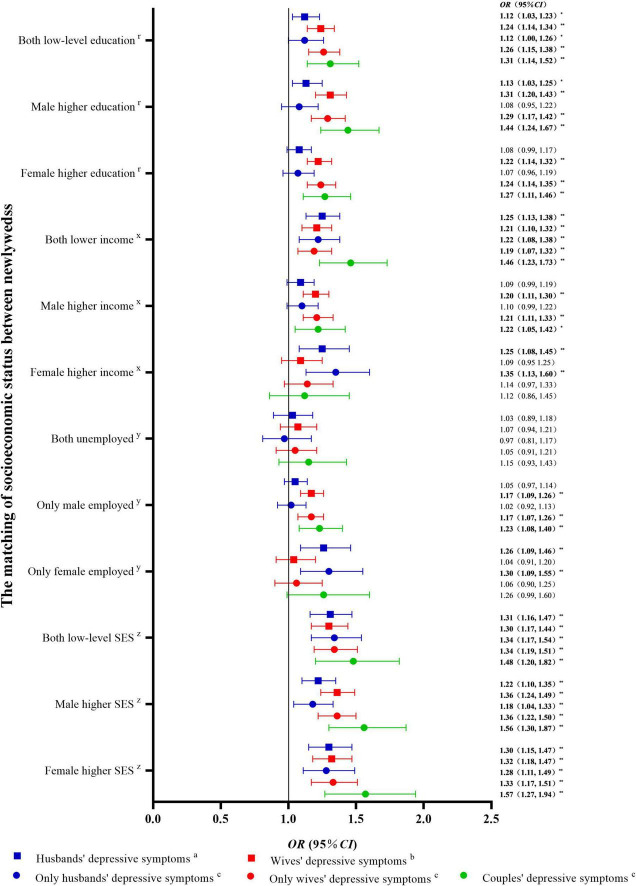
Multivariable logistic regression between socioeconomic status matching and depressive symptoms in newlyweds. OR, odds ratio; CI, confidence interval; SES, socioeconomic status. *^a^*Adjusted for male age, male body mass index (BMI), region, current pregnancy, and male physical activity. *^b^*Adjusted for female age, female BMI, region, current pregnancy, and female physical activity. *^c^*Adjusted for male age, male BMI, region, male physical activity, female age, female BMI, current pregnancy, and female physical activity. ^**^P < 0.01: *P < 0.05. *^r^*compared with newlyweds with both high-level education. *^x^*compared with newlyweds with both high-level incomes. *^y^*compared with newlyweds with both employed. *^z^*compared with newlyweds with both high-level SES.

A multivariable logistic regression model was used with SES matching as the independent variable and depressive symptoms (PHQ-9 score of 0–4 [no] vs. 5–27 [mild or moderate to severe]) as the dependent variables. The results after adjusting for covariates such as age, BMI, region, current pregnancy, and physical activity are shown in Figure 5. Compared with newlyweds with both partners having high-level education or high-level income, newlyweds with low-level education or income homogamy had an increased risk of depressive symptoms in individuals and couples (*P* < 0.05). Furthermore, compared with newlyweds with both high-level education partners, newlyweds with male higher education and female higher education showed an increased risk of depressive symptoms in wives and couples (*P* < 0.05). Compared with newlyweds with both partners having high-level income and employment, newlyweds with male higher income and only husbands being employed had an increased risk of depressive symptoms in wives and couples (*P* < 0.05). The newlyweds with female higher income and only wives being employed had an increased risk of depressive symptoms in husbands (*P* < 0.05). Compared with newlyweds with both high-level SES partners, newlyweds with both low-level SES partners, male higher SES, and female higher SES had an increased risk of depressive symptoms in individuals and couples (*P* < 0.05) (see Figure 5 for *OR* and 95% *CI* values). Similar results were obtained when three classifications of depressive symptoms (PHQ-9 score of 0–4 [no] vs. 5–9 [mild] vs. 10–27 [moderate to severe]) were used as alternative indicators of dependent variables (Supplementary Figure 2).

### Dose-response relationship of depressive symptoms in newlyweds

There were significant positive correlations between husbands and wives for each PHQ-9 item score and the total score (*P* < 0.05) (Supplementary Figure 3).

**FIGURE 6:**
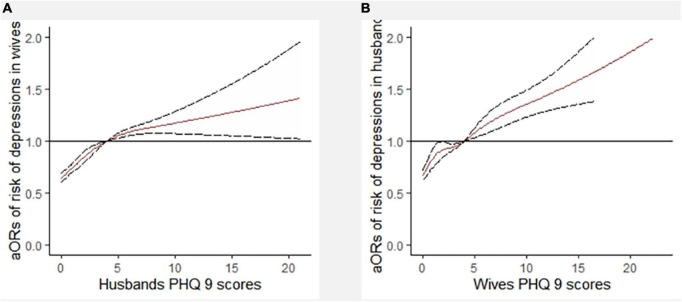
Dose-response relationship between PHQ-9 scores of husbands **(A)** and depression symptoms of wives (6 knots) **(B)**. aOR, adjusted odds ratio; PHQ-9, 9-item Patient Health Questionnaire. Adjusted for male age, male BMI, regions, male physical activity, female age, female BMI, current pregnancy, and female physical activity.

The dose-response association between PHQ-9 scores of husbands and depression symptoms of wives (PHQ-9 score of 0–4 [no] vs. 5–27 [mild or moderate to severe]) was analyzed with the knots at the 5th (0 points), 23rd (0 points), 41st (0 points), 59th (2 points), 77th (4 points), and 95th (8 points) percentile, respectively (Figure 6A). The PHQ-9 score of 4 was used as a reference. After controlling for covariates, when the PHQ-9 scores of husbands reached 4 points, the risk of depressive symptoms in wives was relatively increased. When the PHQ-9 score of the husband was at 0, 2, 4, and 8 points, the risk ratio of the wife for depressive symptoms was 0.64 (0.60, 0.69), 0.84 (0.79, 0.90), 1.00 (1.00, 1.00), and 1.13 (1.07, 1.20), respectively. The dose-response association between PHQ-9 scores of wives and depressive symptoms in husbands (PHQ-9 score of 0–4 [no] vs. 5–27 [mild or moderate to severe]) was investigated with the knots at the 5th (0 points), 23rd (0 points), 41st (1 point), 59th (3 points), 77th (5 points), and 95th (10 points) percentile, respectively (Figure 6B). The PHQ-9 score of 4 was used as a reference. After controlling for covariates, when the PHQ-9 scores of wives reached 4 points, the risk of depression symptoms in husbands was relatively increased. When the PHQ-9 score of the wives was at 0, 1, 3, 5, and 10 points, the risk ratio of the husband for depressive symptoms was 0.66 (0.61, 0.72), 0.84 (0.75, 0.94), 0.94 (0.92, 0.97), 1.09 (1.04, 1.14), and 1.36 (1.23, 1.49), respectively.

**FIGURE 7:**
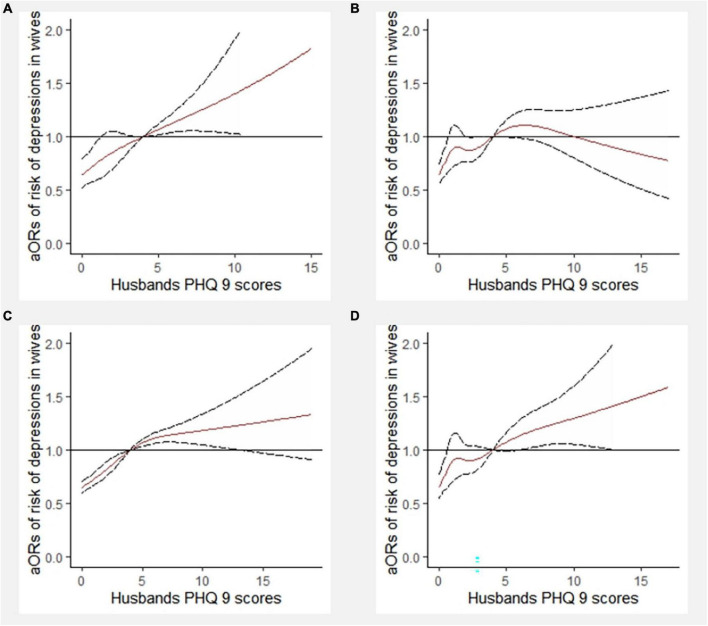
Dose-response relationship between PHQ-9 scores of husbands and depressive symptoms of wives stratified by socioeconomic status (SES) matching (6 knots). aOR, adjusted odds ratio; PHQ-9, 9-item Patient Health Questionnaire. **(A)** In the newlyweds with both high-level SES; **(B)** in the newlyweds with both low-level SES; **(C)** in the newlyweds with male higher SES; **(D)** in the newlyweds with female higher SES.

Furthermore, the dose-response association between PHQ-9 scores of husbands and depressive symptoms of wives, stratified by matching SES, was analyzed using RCS with 6 knots (Figures 7A–D). The PHQ-9 score of 4 was used as a reference. After controlling for covariates, except for newlyweds with both high-level SES partners, there were associations between PHQ-9 scores of husbands and depressive symptoms of wives. In addition, except for newlyweds with both low-level SES partners, there was an increased risk of depressive symptoms in wives when the PHQ-9 scores of husbands reached 4 points. Among newlyweds with both low-level SES partners, the occurrence of depressive symptoms in wives was reduced when husbands had the highest PHQ-9 scores.

**FIGURE 8:**
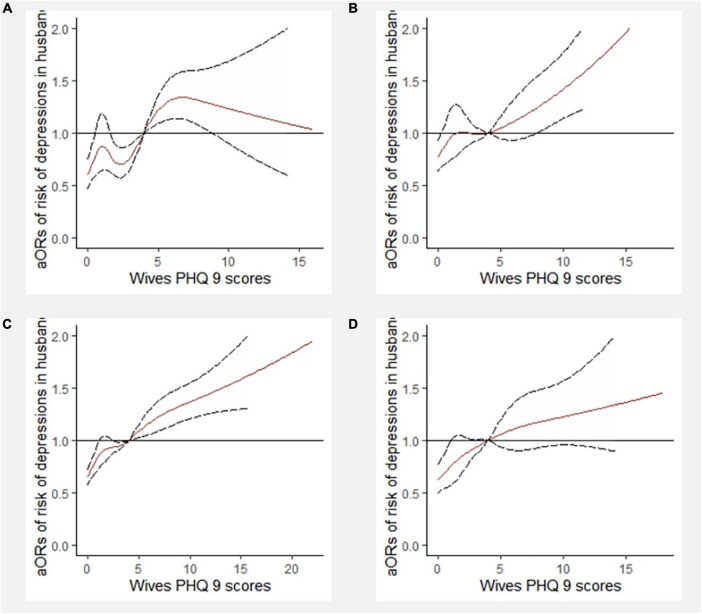
Dose-response relationship between PHQ-9 scores of wives and depressive symptoms of husbands stratified by socioeconomic status (SES) matching (6 knots). aOR, adjusted odds ratio; PHQ-9, 9-item Patient Health Questionnaire. **(A)** In the newlyweds with both high-level SES; **(B)** in the newlyweds with both low-level SES; **(C)** In the newlyweds with male higher SES; **(D)** In the newlyweds with female higher SES.

The dose-response association between the PHQ-9 scores of wives and the depressive symptoms of husbands, stratified by matching SES, was investigated using RCS with 6 knots (Figures 8A–D). The PHQ-9 score of 4 was used as a reference. After controlling for covariates, there were associations between PHQ-9 scores of wives and depressive symptoms of husbands in newlyweds with both high-level SES partners and male higher SES. There was an increased risk of depressive symptoms in wives when the PHQ-9 scores of husbands reached 4 points.

## Discussion

There has been extensive research on SES and depression. However, most studies only considered SES (16–20) for individuals rather than the matching of SES between couples. In the present study, we adopted a couple-centered approach to investigate the effect of SES matching on the co-detection and interaction of depression symptoms in newlyweds based on a large cohort study. We found a mismatch in education, income, and SES to adversely affect depressive symptoms in newlyweds; similar results were obtained for newlyweds with both partners having low-level education, low-level income, or low-level SES. We also found evidence for dose-response associations between depressive symptoms in couples. Compared with previous research, this study provides direct evidence to answer the question of how mismatched SES indicators are associated with the co-detection and interaction of depressive symptoms in newlyweds.

### Association between mismatched SES indicators and depressive symptoms in individuals and couples

Consistent with our hypothesis, we found that newlyweds with mismatched SES had an increased risk of depressive symptoms in individuals and couples. Married couples with matched SES are more likely to have consistent “three views.” Research has found that mismatched SES affects the consistency of domination of family economic and social capital, reducing the space for communication, and increasing the resistance to communication and the emergence of conflict between couples, which seriously affects the marital quality and happiness index of couples (25). These disharmonious family atmospheres can affect the emotional state of couples. In addition, we found that education mismatch in couples, whether male higher education or female higher education, only increases the risk of depressive symptoms in female partners. Similar findings were reported in another Chinese population and in the Composite North Atlantic World (31, 32), which suggested that wives with educational advantages exhibit excessive independent thinking and dominate the family. This weakens the power of the husband in the family and makes him less inclusive in the marriage, which in turn negatively impacts the quality of the marriage. Several theoretical studies have supported the role of communication in marital interactions and the quality of the marital relationship in moderating the connection between spousal depressive symptoms (33). However, this finding is not consistent with other population studies. Based on the 2011 China Health and Retirement Longitudinal Survey data, Lei et al. (34) analyzed a sample of people aged 45 or above and found that women who married someone with a higher SES than themselves had higher life satisfaction and lower levels of depression, but this advantage existed only in rural and less economically developed areas. The study population and the different social situations are a possible explanation for the differences in the results of the different studies.

In addition to the impact of education, another interesting finding of this study is that when one earned less or was unemployed, the risk of depressive symptoms in his or her partner increased. This result is not entirely consistent with previous research. An investigation of the impact of relative household income in the United States on couples showed that when the income of the wife was higher than that of the husband, the marital satisfaction of both parties was lower (35). However, a study from Iran found that married professional women experienced a sharp drop in marital satisfaction when encountered multiple work–family responsibilities and role conflicts (36). Combined with the current social situation, we discuss this result as follows. On the one hand, female or male partners who earn more or are employed will have more power of discourse and autonomy in the family. On the other hand, they may devote more energy to work, have a larger social circle, and are more likely to neglect interactions with their spouse, two factors that could lead to a bad emotional state for their spouse. These findings imply that having a higher income and a stable job are protective factors for depressive symptoms in female partners.

Our findings also provide epidemiological evidence that newlyweds with low-level SES homogamy have an increased risk of depressive symptoms in individuals and couples. These findings are consistent with several epidemiological studies that found an association between low SES and an increased risk of depression (15, 22, 37, 38). In the present study, after adjusting for age, BMI, region, current pregnancy, and physical activity, unemployment in both husbands and wives did not increase the risk of depression, contrary to the impact of education and income level on depressive symptoms in couples. One interpretation is that newlyweds where both husbands and wives are unemployed have more opportunities to communicate, allowing individuals to cope with stressors with more adaptive coping strategies, which is beneficial for their emotional health (39, 40).

### The interplay of depressive symptoms between couples

The present study indicated that depressive symptoms in one partner could affect the other in newlywed couples. It has been shown that perinatal depression in women increases the risk of depression in their husbands (4). In non-pregnant couples, a cross-partner association between depressive symptoms has also been studied (41). Similar results are presented in this study: when the PHQ-9 scores were greater than 4 points, higher depressive symptoms in one partner were associated with higher levels of depression in the other partner. Similar correlations were found in newlyweds with mismatched SES. These findings indicate an increased risk of depressive symptoms in SES-mismatched newlyweds. Furthermore, we found inconsistent results among newlyweds with both low-level SES. A possible explanation for these findings is that differences in marital quality across SES matching indicators can affect their patterns of interaction, leading to different trends in the transmission of depressive symptoms between partners (33).

### Strengths and limitations

The current study has additional strengths that lend confidence to the findings. First, a relatively large sample was selected for analysis according to geographical distribution and economic development characteristics; thus, the data have good representativeness. Second, this study adopts a couple-centered approach, analyzing the effect of SES matching on depressive symptoms between newlyweds. Finally, we investigated the potential correlation between depressive symptoms in husbands and wives and further discussed the interaction of depressive symptoms between couples with different SES levels.

There are also several limitations to the study. Cross-sectional data may be difficult to interpret to determine a causal correlation. Nonetheless, SES was defined by relatively stable indicators, and depressive symptoms were identified based on the mental and emotional status of participants during the previous 2 weeks. Furthermore, demographic information and PHQ-9 scores were obtained through a self-assessment questionnaire, which may lead to reporting bias. However, the PHQ-9 is a self-administered instrument validated for screening, diagnosis, and assessment of the severity of depressive symptoms with good psychometric properties (30) and has been widely used in previous studies (42–44). Moreover, to increase the credibility of the questionnaire, two reverse quality control items were added to the PHQ-9 scale during the survey.

## Conclusion

Overall, the findings of the study provide direct evidence that mismatched SES indicators had adverse effects on depressive symptoms in newlyweds; similar results were obtained for newlyweds with both partners having low-level SES. In addition, this study found that depressive symptoms had a positive association between couples. Therefore, to reduce depressive symptoms in couples, we should make efforts to reduce social inequality and guide couples to narrow the SES gap with equal rights to compete for quality jobs. At the same time, it is equally important to reduce social inequality by increasing access to education, improving the quality of life of people, and enhancing competitiveness in society.

## Data availability statement

The raw data supporting the conclusions of this article will be made available by the authors, without undue reservation.

## Ethics statement

Written informed consent was obtained from the individual(s) for the publication of any potentially identifiable images or data included in this article.

## Author contributions

HG: conceptualization, investigation, data curation and analysis, and writing—original draft preparation and revision. ML, XW, QY, YT, and BW: conceptualization and investigation. KL and PZ: conceptualization, investigation supervision, and writing—reviewing and editing. SS: critical revision and writing—reviewing and editing. FT: project administration and supervision, funding acquisition, critical revision, and writing—reviewing and editing. All authors approved the final version to be published.
