# Supplementary material for: Low and mismatched socioeconomic status between newlyweds increased their risk of depressive symptoms: A multi-center study
Source: Front Psychiatry. 2023 Jan 10;13:1038061. doi: 10.3389/fpsyt.2022.1038061 (PMC9871563; doi:10.3389/fpsyt.2022.1038061)
Supplement: Supplementary file 1 [file Data_Sheet_1.docx]

**Supplementary file**

**Captions**

**Table S1** Differences in the detection rate of depressive symptoms in different sociodemographic characteristics

**Table S2** Distributions of sociodemographic characteristics among the matching of SES indicators

**Figure S1** Unvariable logistic regression between the matching of newlyweds’ socioeconomic status indicators and the degree of depressive symptoms

**Figure S2** Multivariable logistic regression between the matching of newlyweds’ socioeconomic status indicators and the degree of depressive symptoms

**Figure S3** Spearman correlation of the scores of each item of the PHQ-9 scale between newlyweds

| **Table S1 Differences in the detection rate of depressive symptoms in different sociodemographic characteristics** | | | | | | | | | | |
| --- | --- | --- | --- | --- | --- | --- | --- | --- | --- | --- |
|  |  | n | Detection of depression | | *χ2* | *P* value | Degree of depression | | *χ*2 | *P* value |
|  |  |  | C1 (%) | C2 (%) |  |  | C3 (%) | C4 (%) |  |  |
| **Male partners** | | | | | | |  |  |  |  |
| Age of recruitment (year) | | | | | | |  |  |  |  |
|  | ≤23 | 4121 | 79.5 | 20.5 | **5.456** | **0.019^a^** | 16.9 | 3.6 | **-0.032** | **0.019^b^** |
|  | 24～28 | 17507 | 81.1 | 18.9 |  |  | 16.1 | 2.9 |  |  |
|  | ≥29 | 6551 | 81.5 | 18.5 |  |  | 15.6 | 2.9 |  |  |
| Regions | | | | | | |  |  |  |  |
|  | Middle of Anhui | 12981 | 82.4 | 17.6 | **33.729** | **<.001** | 15.0 | 2.6 | **38.599** | **<.001** |
|  | North of Anhui | 7093 | 79.3 | 20.7 |  |  | 17.1 | 3.6 |  |  |
|  | South of Anhui | 8105 | 80.1 | 19.9 |  |  | 16.8 | 3.1 |  |  |
| Current pregnancy status of female partners | | | | | | |  |  |  |  |
|  | Not pregnancy | 21720 | 81.0 | 19.0 | 0.000 | 0.997 | 16.2 | 2.8 | 4.216 | 0.121 |
|  | Pregnancy | 6457 | 81.0 | 19.0 |  |  | 15.7 | 3.3 |  |  |
| Education | | | | | | |  |  |  |  |
|  | Junior high school or below | 5707 | 79.3 | 20.7 | **44.142** | **<.001^a^** | 16.3 | 4.4 | **-0.090** | **<.001^b^** |
|  | High school or technical secondary school | 5623 | 78.9 | 21.1 |  |  | 16.9 | 4.2 |  |  |
|  | College degree or above | 16849 | 82.2 | 17.8 |  |  | 15.7 | 2.1 |  |  |
| Personal annual income(ten thousand yuan) | | | | | | |  |  |  |  |
|  | **<**6 | 8133 | 78.2 | 21.8 | **73.423** | **<.001^a^** | 18.2 | 3.6 | **-0.105** | **<.001^b^** |
|  | 6～10 | 11473 | 81.0 | 19.0 |  |  | 16.0 | 3.0 |  |  |
|  | ≥10 | 8573 | 83.4 | 16.6 |  |  | 14.2 | 2.4 |  |  |
| Employment (last one month) | | | | | | |  |  |  |  |
|  | Employed | 25692 | 81.3 | 18.7 | **17.630** | **<.001** | 15.9 | 2.8 | **24.530** | **<.001** |
|  | Unemployed | 2486 | 77.8 | 22.2 |  |  | 17.9 | 4.3 |  |  |
| Physical activity | | | | | | |  |  |  |  |
|  | High level | 4806 | 83.5 | 16.5 | **208.298** | **<.001** | 14.4 | 2.1 | **259.998** | **<.001** |
|  | Moderate level | 11557 | 84.0 | 16.0 |  |  | 14.1 | 1.9 |  |  |
|  | Low level | 11726 | 77.0 | 23.0 |  |  | 18.6 | 4.4 |  |  |
| BMI | | | | | | |  |  |  |  |
|  | Underweight | 1436 | 79.7 | 20.3 | **11.772** | **0.008** | 16.7 | 3.6 | **14.314** | **0.008** |
|  | Normal weight | 14406 | 81.5 | 18.5 |  |  | 15.7 | 2.8 |  |  |
|  | Overweight | 9718 | 80.9 | 19.1 |  |  | 16.2 | 2.9 |  |  |
|  | Obesity | 2172 | 78.7 | 21.3 |  |  | 17.7 | 3.6 |  |  |
| **Female partners** | | |  |  |  |  |  |  |  |  |
| Age of recruitment (year) | | | | | | |  |  |  |  |
|  | ≤23 | 7200 | 69.1 | 30.9 | **44.462** | **<.001^a^** | 23.0 | 7.9 | **-0.088** | **<.001^b^** |
|  | 24～28 | 17155 | 74.0 | 26.0 |  |  | 21.0 | 5.0 |  |  |
|  | ≥29 | 3824 | 73.8 | 26.2 |  |  | 21.4 | 4.8 |  |  |
| Regions | | | | | | |  |  |  |  |
|  | Middle of Anhui | 12981 | 73.4 | 26.6 | **9.268** | **0.01** | 21.3 | 5.3 | **13.560** | **0.009** |
|  | North of Anhui | 7093 | 71.4 | 28.6 |  |  | 22.2 | 6.4 |  |  |
|  | South of Anhui | 8105 | 72.8 | 27.2 |  |  | 21.6 | 5.6 |  |  |
| Current pregnancy status of female partners | | | | |  |  |  |  |  |  |
|  | Not pregnancy | 22214 | 75.4 | 24.6 | **345.941** | **<.001** | 20.0 | 4.6 | **409.956** | **<.001** |
|  | Pregnancy | 6601 | 63.7 | 36.3 |  |  | 26.9 | 9.4 |  |  |
| Education | | | | | | |  |  |  |  |
|  | Junior high school or below | 5500 | 68.6 | 31.4 | **127.557** | **<.001^a^** | 22.3 | 9.1 | **-0.154** | **<.001^b^** |
|  | High school or technical secondary school | 4195 | 67.5 | 32.5 |  |  | 25.3 | 7.2 |  |  |
|  | College degree or above | 18484 | 75.1 | 24.9 |  |  | 20.6 | 4.3 |  |  |
| Personal annual income(ten thousand yuan) | | | | | | |  |  |  |  |
|  | **<**6 | 19015 | 70.7 | 29.3 | **124.459** | **<.001^a^** | 22.7 | 6.6 | **-0.154** | **<.001^b^** |
|  | 6～10 | 6701 | 76.0 | 24.0 |  |  | 19.9 | 4.1 |  |  |
|  | ≥10 | 2463 | 79.3 | 20.7 |  |  | 17.6 | 3.1 |  |  |
| Employment (last one month) | | | | | | |  |  |  |  |
|  | Employed | 20691 | 74.8 | 25.2 | **166.041** | **<.001** | 20.6 | 4.6 | **227.713** | **<.001** |
|  | Unemployed | 6954 | 66.8 | 33.2 |  |  | 24.5 | 8.5 |  |  |
| Physical activity | | | | | | |  |  |  |  |
|  | High level | 2007 | 75.9 | 24.1 | **181.350** | **<.001** | 20.0 | 4.0 | **202.250** | **<.001** |
|  | Moderate level | 10698 | 76.8 | 23.2 |  |  | 18.9 | 4.3 |  |  |
|  | Low level | 15352 | 69.5 | 30.5 |  |  | 23.6 | 6.9 |  |  |
| BMI | | | | | | |  |  |  |  |
|  | Underweight | 3882 | 72.7 | 27.3 | **8.663** | **0.034** | 21.7 | 5.6 | **37.099** | **<.001** |
|  | Normal weight | 17501 | 73.3 | 26.7 |  |  | 21.5 | 5.2 |  |  |
|  | Overweight | 4258 | 71.7 | 28.3 |  |  | 21.6 | 6.7 |  |  |
|  | Obesity | 2101 | 70.9 | 29.1 |  |  | 21.2 | 7.9 |  |  |
| Abbreviations: BMI = Body Mass Index; SES = socioeconomic status.  Note: C1 - Detection rate of Non-depressive symptoms; C2 - Detection rate of depressive symptoms; C3 - Detection rate of mild depressive symptoms; C4 - Detection rate of moderate to severe depressive symptoms. | | | | | | | | | | |
| ^a^ chi-square test of trend; ^b^ Goodman-kruskal Gamma. | | | | | | |  |  |  |  |

| **Table S2 Distributions of sociodemographic characteristics among the matching of SES indicators** | | | | | | | | | | | | | |
| --- | --- | --- | --- | --- | --- | --- | --- | --- | --- | --- | --- | --- | --- |
|  |  | n | F_M_education | |  | F_M_income | |  | F_M_employment | |  | F_M_SES | |
|  |  |  | *χ*2 value | *P* value |  | *χ*2 value | *P* value |  | *χ*2 value | *P* value |  | *χ*2 value | *P* value |
| Regions |  |  |  |  |  |  |  |  |  |  |  |  |  |
|  | Middle of Anhui | 13274 | **757.704** | **<.001** |  | **394.510** | **<.001** |  | **542.866** | **<.001** |  | **198.931** | **<.001** |
|  | North of Anhui | 7345 |  |  |  |  |  |  |  |  |  |  |  |
|  | South of Anhui | 8198 |  |  |  |  |  |  |  |  |  |  |  |
| **Female** |  |  |  |  |  |  |  |  |  |  |  |  |  |
| Age of recruitment (year) | | | | | | | | | |  |  |  |  |
|  | ≤ 23 | 7473 | **3122.001** | **<.001** |  | **1389.743** | **<.001** |  | **1478.849** | **<.001** |  | **905.286** | **<.001** |
|  | 24～28 | 17447 |  |  |  |  |  |  |  |  |  |  |  |
|  | ≥ 29 | 3897 |  |  |  |  |  |  |  |  |  |  |  |
| Physical activity | | | | | | |  |  |  |  |  |  |  |
|  | High level | 2059 | **369.290** | **<.001** |  | **95.089** | **<.001** |  | **45.353** | **<.001** |  | **88.390** | **<.001** |
|  | Moderate level | 10899 |  |  |  |  |  |  |  |  |  |  |  |
|  | Low level | 15707 |  |  |  |  |  |  |  |  |  |  |  |
| BMI |  |  |  |  |  |  |  |  |  |  |  |  |  |
|  | Underweight | 3961 | **1016.162** | **<.001** |  | **243.665** | **<.001** |  | **477.690** | **<.001** |  | **187.596** | **<.001** |
|  | Normal weight | 17842 |  |  |  |  |  |  |  |  |  |  |  |
|  | Overweight | 4354 |  |  |  |  |  |  |  |  |  |  |  |
|  | Obesity | 2161 |  |  |  |  |  |  |  |  |  |  |  |
| **Male** |  |  |  |  |  |  |  |  |  |  |  |  |  |
| Age of recruitment (year) | | | |  |  |  |  |  |  |  |  |  |  |
|  | ≤ 23 | 4227 | **1730.939** | **<.001** |  | **1001.671** | **<.001** |  | **826.104** | **<.001** |  | **593.272** | **<.001** |
|  | 24～28 | 17827 |  |  |  |  |  |  |  |  |  |  |  |
|  | ≥ 29 | 6713 |  |  |  |  |  |  |  |  |  |  |  |
| Physical activity | |  |  |  |  |  |  |  |  |  |  |  |  |
|  | High level | 4897 | **373.538** | **<.001** |  | **112.323** | **<.001** |  | **76.676** | **<.001** |  | **132.214** | **<.001** |
|  | Moderate level | 11750 |  |  |  |  |  |  |  |  |  |  |  |
|  | Low level | 12006 |  |  |  |  |  |  |  |  |  |  |  |
| BMI |  |  |  |  |  |  |  |  |  |  |  |  |  |
|  | Underweight | 1463 | **167.984** | **<.001** |  | **74.687** | **<.001** |  | **98.525** | **<.001** |  | **55.450** | **<.001** |
|  | Normal weight | 14698 |  |  |  |  |  |  |  |  |  |  |  |
|  | Overweight | 9889 |  |  |  |  |  |  |  |  |  |  |  |
|  | Obesity | 2210 |  |  |  |  |  |  |  |  |  |  |  |
| Abbreviations: BMI = Body Mass Index; SES = socioeconomic status; F_M_education = Educational differences between couples; F_M_income = Income differences between couples; F_M_employment = employment differences between couples; F_M_SES = socioeconomic status differences between couples | | | | | | | | | | | | | |


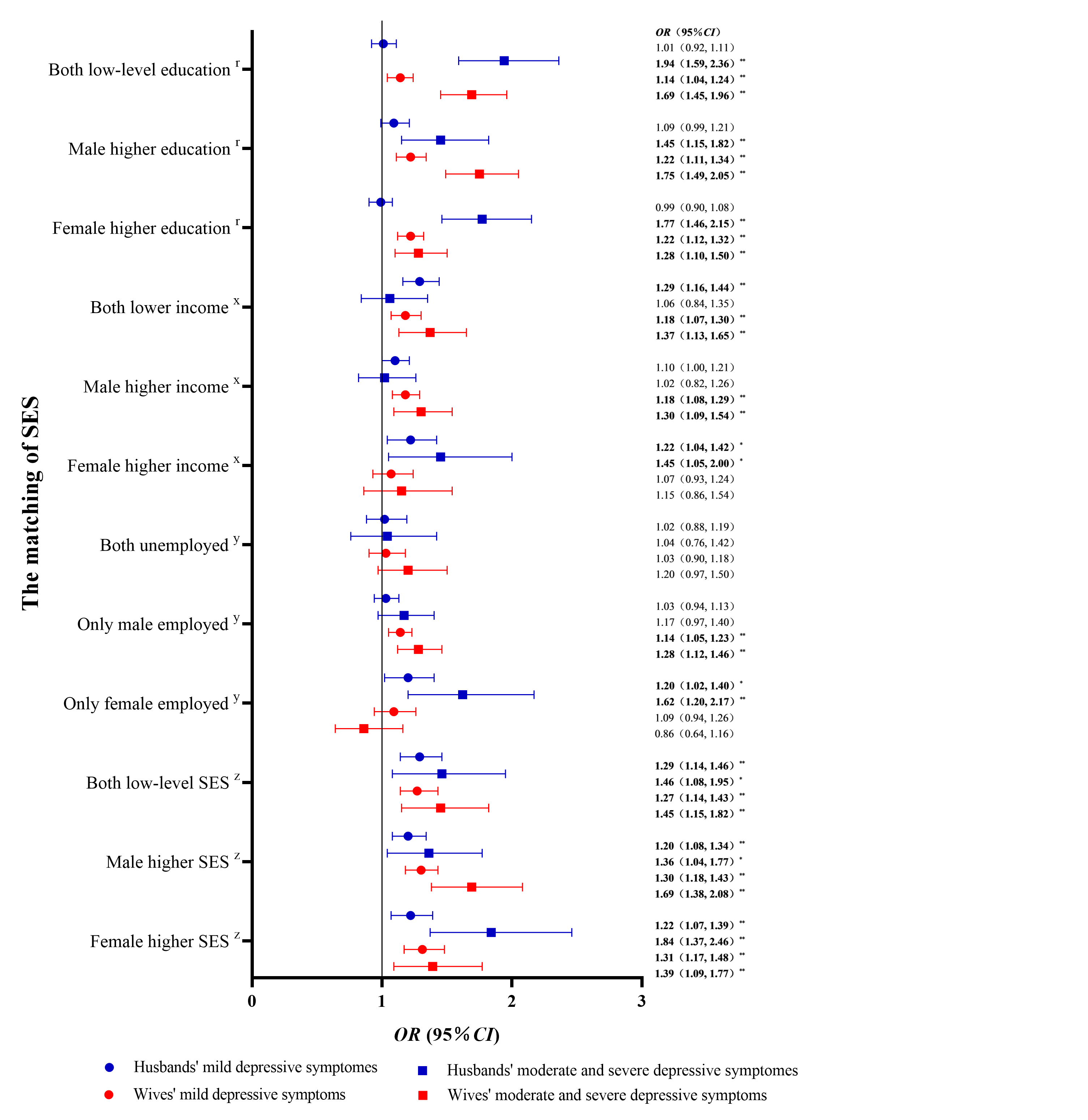


**Figure S1 Unvariable logistic regression between the matching of newlyweds’ socioeconomic status indicators and the degree of depressive symptoms**

Abbreviations: *OR* = odds ratio; *CI* = confidence interval; SES = socioeconomic status.

Note: ^**^ *= P*<0.01; ^*^ = *P*<0.05.

^r^ compared with newlyweds with both high-level education.

^x^ compared with newlyweds with both high-level income.

^y^ compared with newlyweds with both employed.

^z^ compared with newlyweds with both high-level SES.


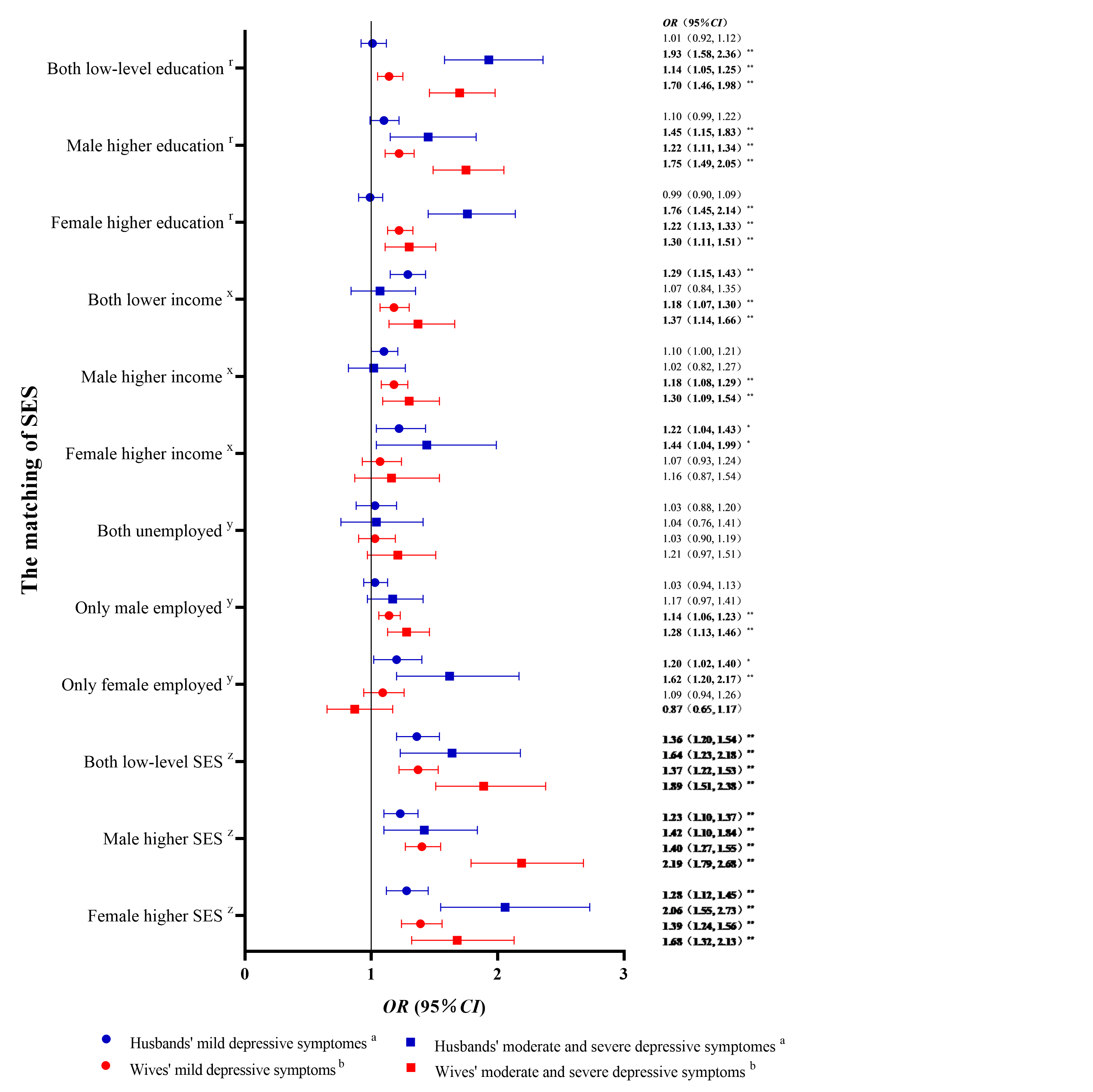


**Figure S2 Multivariable logistic regression between the matching of newlyweds’ socioeconomic status indicators and the degree of depressive symptoms**

Abbreviations: *OR* = odds ratio; *CI* = confidence interval; SES = socioeconomic status.

Note: ^a^ adjusted for male age, male BMI, region, current pregnancy, and male physical activity.

^b^ adjusted for female age, female BMI, region, current pregnancy, and female physical activity.

^**^ *= P*<0.01; ^*^ *= P*<0.05.

^r^ compared with newlyweds with both high-level education.

^x^ compared with newlyweds with both high-level income.

^y^ compared with newlyweds with both employed.

^z^ compared with newlyweds with both high-level SES.


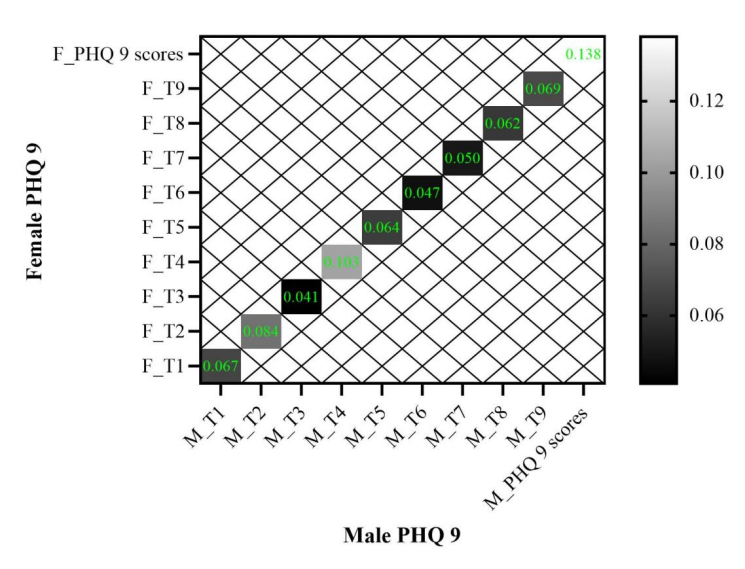


**Figure S3 Spearman correlation of the scores of each item of the PHQ-9 scale between newlyweds**

Abbreviations: F = Females; M = males; PHQ-9 = the 9-item Patient Health Questionnaire.

Note: T1 - Do you have no energy or interest in doing things? T2 - Feeling low, depressed, or hopeless? T3 - Difficulty falling asleep, restless sleep or excessive sleep? T4 - Feeling tired or unenergized? T5 - Loss of appetite or eating too much? T6 - Feel bad about yourself or a failure, or let yourself or your family down? T7 - Have trouble focusing on things (such as reading a newspaper or watching TV)? T8 - Move or speak slowly enough for others to notice? Or is it the other way around, fidgeting or fidgeting more than usual? T9 - Have thoughts of dying or hurting yourself in some way?
